# Impact of Patient Online Record Access on Documentation: Scoping Review

**DOI:** 10.2196/64762

**Published:** 2025-02-20

**Authors:** Eva Meier-Diedrich, Camilla Lyckblad, Gail Davidge, Maria Hägglund, Anna Kharko, Brian McMillan, Charlotte Blease, Julian Schwarz

**Affiliations:** 1 Department of Psychiatry and Psychotherapy Center for Mental Health, Immanuel Hospital Rüdersdorf Brandenburg Medical School Theodor Fontane Rüdersdorf Germany; 2 Faculty of Health Sciences Brandenburg Brandenburg Medical School Theodor Fontane Neuruppin Germany; 3 Department of Archives, Libraries and Museums Uppsala University Uppsala Sweden; 4 Centre for Medical Humanities Uppsala University Uppsala Sweden; 5 Centre for Primary Care and Health Services Research University of Manchester Manchester United Kingdom; 6 Participatory eHealth and Health Data Research Group Department of Women's and Children's Health Uppsala University Uppsala Sweden; 7 Uppsala University Hospital Uppsala Sweden

**Keywords:** open notes, electronic health record, open record access, clinical notes, eHealth

## Abstract

**Background:**

Online record access (ORA) is being increasingly implemented internationally. Despite reported benefits for patients, health care professionals (HCPs) have raised concerns about potential disadvantages. To date, no review has examined the empirical evidence on whether and how documentation changes following the introduction of patients’ ORA.

**Objective:**

This scoping review aimed to examine potential subjective and objective changes in HCPs’ documentation after using patients’ ORA.

**Methods:**

A scoping review was conducted using a methodological framework for scoping reviews and data from 4 electronic databases. Studies examining objective and subjective changes in clinical documentation following the implementation of ORA, specifically those related to actual use experiences (rather than previous expectations), up to July 2023, were included. We used the Mixed Methods Appraisal Tool to assess the quality of the included studies. The narrative synthesis and reporting of findings were guided by the PRISMA-ScR (Preferred Reporting Items for Systematic Reviews and Meta-Analyses Extension for Scoping Reviews).

**Results:**

Of the 3143 papers screened, 42 (1.34%) were included in this review. The included studies mainly used qualitative methods and were predominantly published after 2016 in the United States. The included studies were conducted in different settings (inpatient and outpatient) and clinical areas (somatic, mental health, and other). In total, 8 studies analyzed clinical notes, while the remaining studies focused on the experiences of patients, HCPs, and other stakeholders with ORA. Objectively, a decrease in complexity, an increase in readability, and a change in the emotional tone of the clinical notes were observed. The length of the clinical notes was observed to change both objectively and subjectively, although the direction of this change was inconclusive. However, many HCPs also reported writing notes that were less open and more restrictive to protect sensitive or hypothetical information. While for some HCPs the implementation of ORA made the clinical notes a less efficient and valuable working tool, others perceived that ORA opened up new therapeutic opportunities through direct contact with patients.

**Conclusions:**

The question of whether an inherently uniform clinical note can meet the diverse needs of different health care stakeholders remains unresolved, highlighting the challenges of standardizing practices in this complex sector. While ORA may encourage HCPs to make their clinical notes more patient friendly, it may also compromise the integrity of documentation by omitting sensitive findings and expert judgment, which can put patients at risk and lead to errors that increase the risk of malpractice. Given the limitations of digital documentation in fostering trust, it is imperative to prioritize meaningful patient-HCP interactions. The use of compensatory measures, such as parallel documentation and restricted access to clinical notes, indicates systemic problems and suggests that current practices are suboptimal.

**International Registered Report Identifier (IRRID):**

RR2-10.2196/46722

## Introduction

### Background

The electronic health record (EHR) has evolved over time. Initially designed as a memory aid for physicians, a communication tool among clinicians, and a system for billing and reimbursement, it has more recently been made accessible to patients [1,2]. In a growing number of countries, patients are now granted partial or full access to their EHRs [3-7]. The Nordic countries and the United States have been at the forefront of this movement [8-10]. A crucial component of patients’ online record access (ORA) involves accessing the clinical free-text notes written by clinicians. Granting patients access to these notes is commonly referred to as “open notes” in the literature [3]. Patient ORA reflects the zeitgeist of greater transparency in societal institutions and health care [11]. Several benefits have been cited as motivations for this practice, including empowering patients through transparency and access to information, as well as fostering innovation within the health application economy [8,12].

While ORA fulfills patient demand for transparency in care, it also necessitates a cultural shift toward higher degrees of openness among institutions and HCPs, possibly acting as a disrupter in information management behaviors among the clinicians creating them. Previous research indicates that health care professionals (HCPs) have frequently expressed skepticism toward patients’ ORA [13-15]. HCPs have voiced concerns about increased workload, changed clinical routines, and ORA impacting patient safety and privacy [1,13,14,16]. Some have expressed concerns over spending more time writing notes and addressing patient inquiries and also anticipated confusion and offense among patients, particularly regarding mental health issues [3]. In terms of documentation, many HCPs expected to alter both the content and tone of their notes when patients have ORA, indicating that the practice could potentially undermine the integrity of their records [4,5,17]. For instance, a tendency to avoid technical terminology and medical jargon to enhance patient comprehension might detrimentally affect multidisciplinary communication within the team [5,18]. Other HCPs worried that they may become less detailed or candid in their documentation, feeling the need to omit information or resort to parallel documentation (a so-called shadow record) to shield patients from potentially harmful or disruptive information [13,14,19,20]. An often-overlooked risk of ORA is that individuals considered vulnerable may be pressured into revealing their records to third parties, such as relatives or abusers, potentially leading to unauthorized access to sensitive patient information without consent [13,14,19,20]. However, it is also possible that ORA could make notes more patient friendly by encouraging clinicians to use more patient-centered, more understandable, and less stigmatizing language while stimulating communication between HCPs and patients [4,21].

Studies investigating the impact of ORA on clinical documentation have predominantly concentrated on the patient experience, with limited research examining the HCPs’ perspective [22]. As noted by Blease et al [1], while these studies investigate self-reports about possible documentation changes, few studies have focused on objective changes following implementation. Where such studies exist, they appear to offer inconclusive results [4,23,24] and are frequently hindered by methodological limitations. There is a growing body of qualitative research [25], along with research using natural language processing methods to examine the language used by clinicians in their records, including the potential for stigmatizing language [22]. However, it remains unclear from these studies whether patient ORA influences or indeed enhances the quality of record keeping, given the awareness that patients may read the clinician’s notes [22,26].

### Objectives

As highlighted earlier, HCPs are often reluctant or critical toward granting patients ORA and anticipate an additional documentation burden upon its introduction. Therefore, this scoping review focuses exclusively on studies containing postimplementation data, encompassing the experiences of various stakeholders, such as patients, HCPs, and other health care providers, while excluding preimplementation expectations. This study seeks to (1) identify, compile, and assess reported objective and subjective changes in documentation following the implementation of ORA; (2) enhance stakeholders’ knowledge of the types of documentation changes that may arise because of ORA policy implementation; (3) highlight implications for documentation practices and offer recommendations for improving future clinical practice; and (4) identify knowledge gaps warranting further research.

## Methods

### Scoping Review

Compared to the systematic review method, which is guided by a strongly focused research question, a scoping review aims to broaden the spectrum of the available evidence in a relatively new field of research, allowing its breadth and depth to be clearly seen [27]. We conducted a scoping review following the framework proposed by Arksey and O’Malley [27]. Their approach consists of the following five stages: (1) identifying the research question, (2) identifying the relevant studies, (3) selecting eligible studies, (4) collecting data, and (5) summarizing data and synthesizing results. The review is reported following the PRISMA-ScR (Preferred Reporting Items for Systematic Reviews and Meta-Analyses Extension for Scoping Reviews) checklist (Multimedia Appendix 1) [28,29]. We adhered closely to the methodological approach outlined in our published review protocol [30]. Any minor deviations are comprehensively described.

### Stage 1: Identifying the Research Question

Through discussions with the research team, we decided on the following research questions: (1) Does clinical documentation change after introducing ORA for patients? and (2) If so, what objective and subjective changes arise after ORA implementation? By objective, we mean the differences that can be demonstrated by a direct, quantifiable comparison of clinical notes before and after the implementation of ORA. By subjective, we refer to clinicians’ perceptions of how they write their notes after ORA implementation. In the context of this scoping review, we define ORA as any channel in which patients have electronic access to their patient records (eg, through the internet and via tethered patient portals and apps).

### Stage 2: Identifying Relevant Studies

First, the research team performed a rigorous manual search to obtain a basic overview of the available evidence and to refine the scope of the review as well as the search strategy, as suggested by Popay et al [31]. The literature search was then conducted in the following 4 databases on July 31, 2023: APA PsycINFO, CINAHL, PubMed, and Web of Science Core Collection. The deduplication process was then performed. The search strategy consisted of three key concepts: (1) EHRs, (2) sharing EHRs with patients, and (3) changes in documentation, which were combined with the Boolean “AND” (Textbox 1). The search terms were adapted according to different databases. The complete search string can be found in Multimedia Appendix 2.

**Key concepts of the search strategy.**

**Search string for the electronic health record**

- “inpatient portal*” OR “open notes” OR opennotes OR PAEHR OR “patient portal*” OR “patient web portal*” OR “Electronic Health Records”
- “clinic notes” OR “clinical notes” OR “progress notes” OR “doctors notes” OR EHR OR “health record*” OR “health care record*” OR “medical record*” OR “mental health notes” OR “patient record*” OR “psychiatric notes” OR “psychotherapy notes” OR “visit notes”

**Search string for sharing the electronic health records with patients**

- “guardian access” OR “parental access” OR “parents access” OR “patient access*” OR “patients access*” OR “patient online access” OR “patients online access” OR “proxy access” OR “shared medical record*” OR “shared health record*”

**Search string for documentation changes**

- “Language”[Mesh] OR “Attitude”[Mesh] OR “Comprehension”[Mesh]
- accura* OR ambigu* OR characteristics OR characters OR clarity OR content* OR completeness OR comprehend* OR comprehensibl* OR comprehension* OR correctness OR dialog* OR express* OR directness OR impression* OR inaccura* OR incomplete* OR incomprehen* OR incorrectness* OR intelligib* OR interpret* OR intuitive* OR language OR length OR linguistic* OR misconception* OR misinterpret* OR misread* OR misunderstand* OR monolog* OR negative* OR pattern* OR positive* OR pronoun* OR readab* OR style* OR simplicity OR terminolog* OR transparen* OR truthful* OR unambigu* OR understand* OR untruthful* OR veracity OR wordcount* OR words OR writing
- OR attitude* OR emotion* OR experience* OR perception* OR satisfact*
- OR adopt* OR alter* OR censor* OR change* OR changing OR difference* OR introduc* OR implement* OR modif* OR postimplement*

### Stage 3: Selecting Eligible Studies

#### Inclusion and Exclusion Criteria

Inclusion and exclusion criteria were defined by the entire research team and were applied in the study selection process (Textbox 2). Due to the limited number of publications available on the subject, there were no restrictions on the study type. As ORA is only gradually being implemented in various countries, we refrained from any location restrictions. A wide variety of approaches exist to make clinical notes available to patients electronically [32]. We included all studies examining the actual implementation and the use of patient ORA regardless of the platform (eg, web browser or mobile apps). Studies that explored the sharing of hard copies of patients’ clinical records were excluded.

During the review process, we refined the inclusion and exclusion criteria as follows: we required studies to provide empirical data on changes in clinical documentation resulting from ORA. Studies that solely focused on secure messaging between patients and clinicians were excluded.

**Inclusion and exclusion criteria.**

**Inclusion criteria**

- Study design: all study types
- Publication: original, peer-reviewed work including empirical data published between January 1, 2005, and June 30, 2023, in English
- Study location: all medical disciplines, all health care settings, and no location restrictions
- Study participants: patients and health care professionals of all ages
- Studies that examine actual use by stakeholders and their experiences with patient-accessible electronic health records
- Studies that provide empirical data on documentation changes resulting from the use of online record access

**Exclusion criteria**

- Paper-based, disc, or USB sharing of patients’ records
- Articles without empirical data (eg, comments, editorials, and news)
- Gray data (websites, tweets, and blogs)
- Studies that exclusively investigate expectations about patient-accessible electronic health records
- Studies on secure messaging

#### Study Selection Process

We used Rayyan Software (Rayyan Systems, Inc) for conducting a collaborative, single-blinded title and abstract screening following the predefined eligibility criteria [33]. All research team members participated in this process, and at least 2 people evaluated each record of the result set. Discrepancies were discussed, taking the full texts of the corresponding studies into account. In case of disagreements that could not be resolved, a third reviewer was involved and entrusted with the decision of including or excluding the study.

### Stage 4: Collecting Data

After selecting the studies to include, metadata (eg, title, authors, and publication year) of the remaining records were exported and summarized in a Google Sheets (Google LLC) spreadsheet for further processing. To extract and organize relevant data from included studies, the spreadsheet was extended by the following parameters based on the studies’ full text: country, study design, sample, characteristics of study participants (eg, gender, age, ethnicity, and type of stakeholder), treatment setting and medical specialty, study purpose, and relevant results (Multimedia Appendix 3). Data extraction was performed by EM-D and checked for correctness and completeness by CL. The quality and methodological rigor of the studies were assessed using the Mixed Methods Appraisal Tool (MMAT) [34]. Two reviewers (CL and EM-D) independently conducted the MMAT grading of all studies and reached consensus concerning the methodological quality of the studies (Multimedia Appendix 4). Two additional researchers (AK and JS) validated the MMAT grading for correctness.

### Stage 5: Summarizing Data and Synthesizing Results

Study results were extracted from the full texts by the lead author (EM-D) and summarized in (1) a reduced format within a textbox, providing an overview of the findings from all included studies; and (2) a detailed version for narrative synthesis. The latter was analyzed independently by 2 researchers (CL and EM-D) using thematic analysis [35]. Objective and subjective changes in HCPs’ documentation practices after the introduction of patient ORA served as guiding deductive themes, informed by the research question. However, they were open to modifications during the analytic process. As suggested by Levac et al [28], we aimed to identify patterns and relationships within and across studies to identify potential factors influencing documentation after ORA implementation. In assessing the methodological rigor of the studies, we also envisaged the potential to identify research gaps; for example, we predicted that there may be a preponderance of survey research investigating clinicians’ perceptions about documentation changes rather than studies investigating objective markers of any such documentation changes. While the former studies may be useful, they may be compromised by responder biases. Results were discussed and approved by the entire research team.

### Ethical Considerations

This study did not require ethics approval, as it only used publicly available data and followed the scoping review methodology.

## Results

### Study Selection

**Figure 1:**
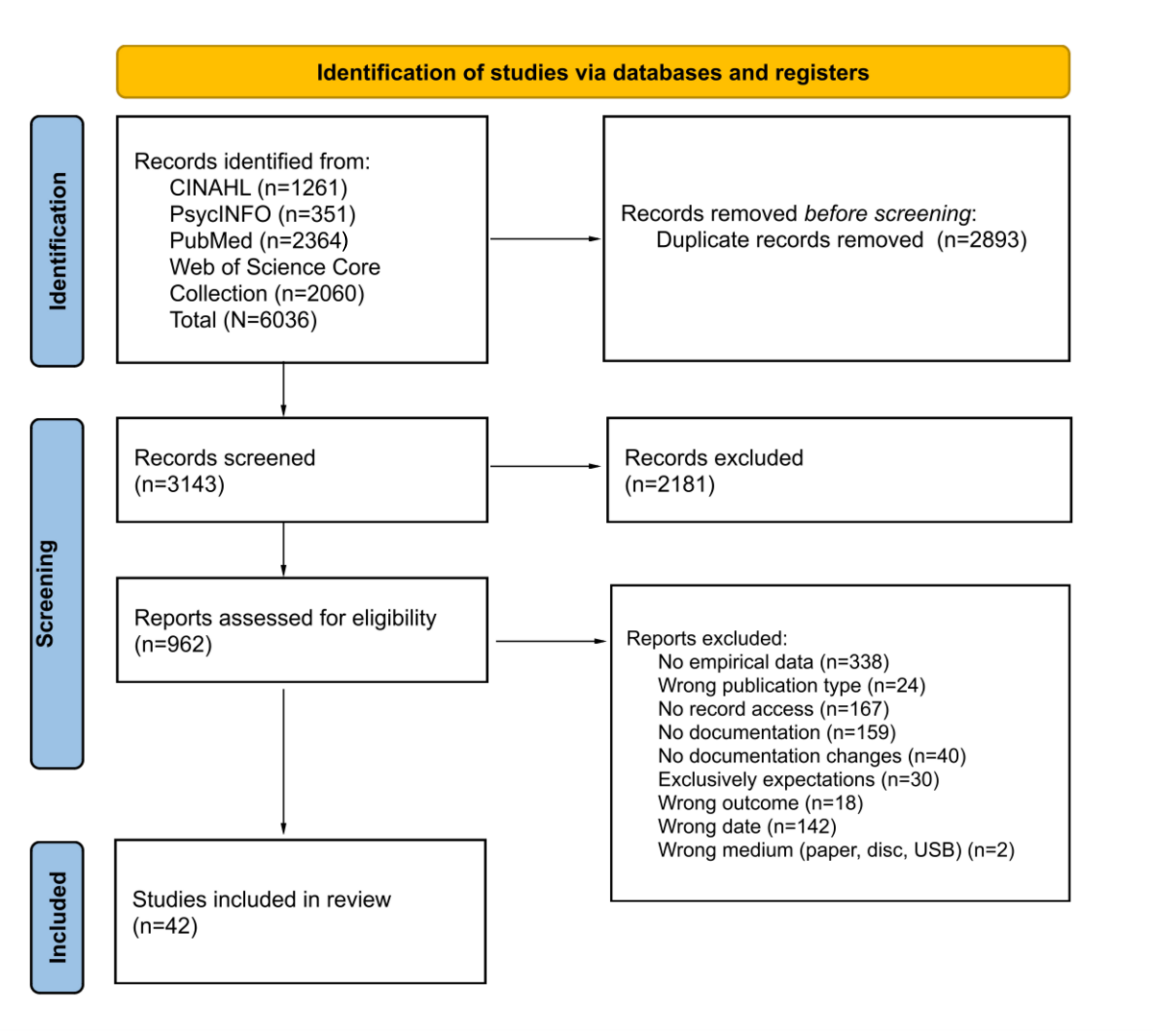
PRISMA (Preferred Reporting Item for Systematic Reviews and Meta-Analyses) flow diagram for study inclusion. ORA: online record access.

A total of 6036 records were identified: 1261 (20.89%) from CINAHL, 351 (5.82%) from PsycINFO, 2364 (39.17%) from PubMed, and 2060 (34.12%) from the Web of Science Core Collection. After removing duplicates, 52.07% (3143/6036) of the records remained for title, abstract, and keyword screening. At this stage, an additional 36.41% (2198/6036) of the records were eliminated, leaving 15.66% (945/6036) of the records for full-text screening to check eligibility. During the full-text screening, 903 studies were excluded, resulting in a final selection of 42 studies that met the inclusion criteria and could be included in the review. The PRISMA (Preferred Reporting Item for Systematic Reviews and Meta-Analyses) flow diagram (Figure 1), adapted from the study by Page et al [36], provides a detailed representation of the study selection process.

### Basic Characteristics of the Body of Evidence

The included studies mainly used qualitative methods, but quantitative, descriptive, and mixed methods were also frequent. Of the 42 studies included, 23 (55%) were conducted in the United States, followed by Sweden with 9 studies (21%). Since 2017, the number of articles published on the topic of documentation changes following ORA implementation has remained relatively constant at 4 to 6 relevant articles per year. Only 19% (8/42) of the included studies analyzed clinical notes and reported on objective documentation changes, while most studies (34/42, 81%) investigated subjective documentation changes. A comprehensive overview of the basic parameters of the included studies can be found in Table 1.

**Table 1:** Basic characteristics of the included studies (N=42).

Parameter				Studies, n (%)		References
**Study design**						
	Quantitative descriptive			11 (26)		[14,23,24,37-44]
	Quantitative nonrandomized			5 (12)		[23,45-48]
	Mixed methods			10 (24)		[4,19,49-56]
	Qualitative			16 (38)		[3,13,17,20,57-68]
**Publication year**						
	2011-2016			8 (19)		[14,37,38,44,47,48,57,63]
	2017-2020			18 (43)		[3,4,39,40,43,49,51,52,55,58,60-62,64,67]
	2021-2024			16 (38)		[13,17,20,23,41,42,45,46,50,53,54,56,59,65,68,69]
**Country**						
	Canada			1 (2)		[52]
	Netherlands			1 (2)		[41]
	Norway			3 (7)		[51,67,68]
	Sweden			9 (21)		[19,37,43,55,58,62-65]
	United Kingdom			5 (12)		[13,17,20,59,61]
	United States			23 (55)		[3,4,14,23,24,38-40,42,44-50,53,54,56,57,60,66,69]
**Participants^a^**						
	Health care professionals			40 (95)		All, except for [40,47]
	Patients			9 (21)		[20,38,44,45,48,54,57,59,66]
	Care partners			3 (7)		[52,56,59]
Studies analyzing clinical notes				8 (19)		[4,23,24,40,45,47,49,53]
**Treatment setting^a^**						
	Inpatient			10 (24)		[38,51,52,55,56,59,60,62,66,67]
	Outpatient			29 (69)		[3,4,14,17,20,23,38,39,43-49,52,55,57,58,60-62,64-69]
	Not specified			10 (24)		[19,24,37,40,42,50,53,54,63]
**Clinical field^a^**						
	Mental health care			9 (21)		[3,14,19,51,60,62,65,66,68]
	**Somatic**					
		Primary	13 (31)		[13,20,38,44-46,53,55,57,58,65,67,69]	
		Oncology	8 (19)		[4,23,24,40,43,49,63,64]	
		General	3 (7)		[17,41,61]	
		Other specialities	5 (12)		[37,48,50,56,59]	
	Multispecialty			6 (14)		[39,42,47,51,52,63]

^a^Individual articles can be assigned to the various subparameters at the same time, which means that percentages >100% can be achieved.

### Search Results

**Figure 2:**
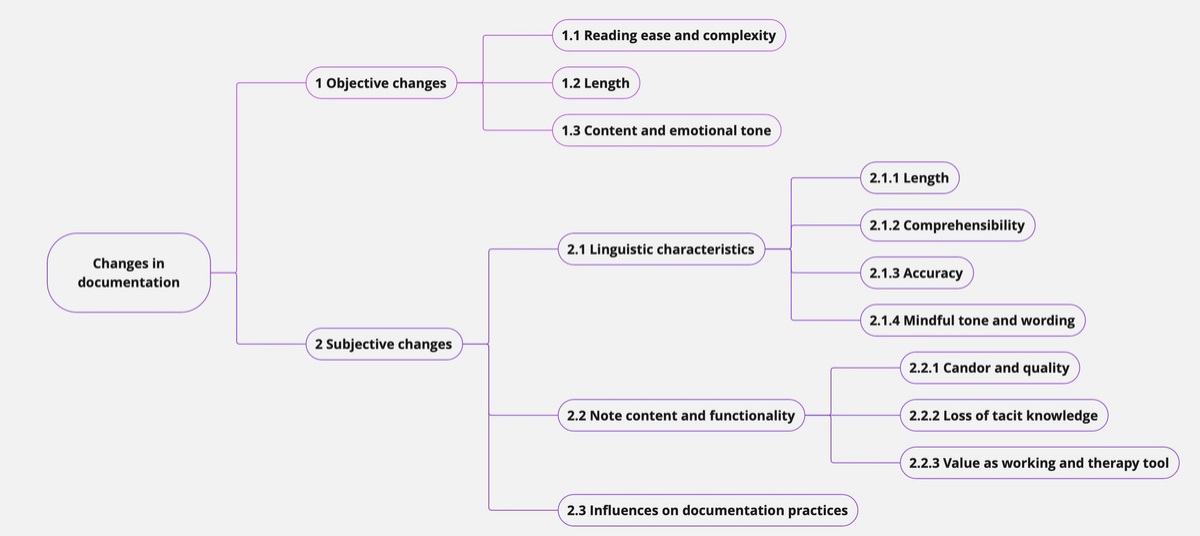
Graphic overview of the objective and subjective changes in documentation.

While several studies specifically examined documentation changes due to ORA implementation (11/42, 26%) [23,24,40,43,45-47,49,53,67,68], many more reported them as a secondary outcome (31/42, 74%). The results were divided into three groups: (1) objective changes, (2) subjective changes, and (3) influences on documentation practices. Both objective and subjective changes and a lack of changes were observed in the included studies. The categories identified for subjective changes were note characteristics, changes in content and functionality, and absence of subjective changes. Figure 2 provides a visual summary of the objective and subjective changes identified in the documentation. For documentation behaviors, the categories identified were influence on writing practices, secure information, and influence of sociodemographics.

### Objective Changes in Documentation

#### Overview

Of the 42 studies we reviewed, 8 (19%) examined clinical notes and 7 (17%) reported objective changes in documentation due to ORA. The number of notes analyzed varied notably, spanning from 200 [4] to 164,806 [53]. The studies used validated scales and tools such as Linguistic Inquiry and Word Count, Flesch-Kincaid Grade Level, Flesch Reading Ease Scores, Gunning Fog Index, Measure of Gobbledygook, Coleman-Liau Index, and Automated Readability Index as outcome measures, alongside other metrics such as the number and frequency of abbreviations, word frequencies, co-occurrences between frequent words, and note length in characters. Most studies (1/15, 7%) found changes in clinical notes after the implementation of ORA; however, there were also results where clinical notes remained unaffected, often for the same outcome measures.

#### Reading Ease and Complexity

In total, 3 studies examined changes in the reading ease and complexity of clinical notes [23,45,47]. Blok et al [45] and Kind et al [47] reported a decrease in reading ease and an increase in complexity, while Rahimian et al [23] found the opposite trend: a significant decrease in complexity and an increase in readability. Changes in reading ease were primarily observed in the clinical progress notes. In contrast, other note types, such as the initial notes and letters [23] or the assessment and plan sections [47], seemingly did not change.

Both reading ease and complexity are affected by the use of acronyms and clinical abbreviations [45,47]. While Blok et al [45] found no objective changes in the number and frequency of abbreviations used before and after ORA implementation, Kind et al [47] reported a significant increase in the rate of acronyms and abbreviations in clinical notes. However, the absolute rate of acronyms and abbreviations remained low at approximately 3%.

#### Length

When discussing the readability and complexity of the notes, studies often refer to note length as a relevant moderator. Four of the included studies [23,24,45,46] examined objective changes in note length because of ORA implementation. Two studies found a statistically significant increase in the average length of progress notes [23,45]. In contrast, Holmgren and Apathy [46] observed a brief, nonsignificant increase in note length of 27.3 characters after ORA implementation, followed by a statistically significant downward trend in note characters over the long term. These changes mainly appear to apply to unstructured visit notes and are not present in the medical history or physician’s letters [23,45]. In addition, Rahimian et al [24] used n-grams to identify provider-specific variation in note length. N-grams (linguistics) are sequences of “n” items from a given sample of text or speech, where the items can be phonemes, syllables, letters, words, or base pairs, depending on the application. Rahimian et al [24] found that the number of significant n-grams remained consistent across providers.

#### Content and Emotional Tone

When it comes to the content of clinical notes, the objective evidence is inconclusive. Typically, only small to moderate changes in the content of clinical notes are reported when shared with patients via web-based patient portals [40,47]. Rahimian et al [24] and Jain et al [40] both use visual graphing of words used and their relatedness to analyze objective content changes in clinical notes pre- and post-ORA: Before ORA, words such as “follow,” “well,” and “disease” were most prominent, while after ORA, the words “old” and “well” became more prevalent [40]. According to Rahimian et al [24], the use of words such as “distress,” “concerning for,” and “follow-up” decreased in clinical notes post-ORA, while the word “improving” became more prevalent after ORA was implemented. According to an analysis by Alpert et al [49] using Linguistic Inquiry and Word Count, the emotional tone of the notes remained unchanged. With regard to mental health issues, an increase (pulmonology) or decrease (rheumatology) in notes mentioning mental health status was observed depending on the medical department [47].

### Subjective Changes in Documentation

#### Overview

The included studies with qualitative survey components (ie, pure qualitative or mixed methods studies) primarily used qualitative interviews for data collection (18/26, 69%), followed by qualitative analysis of written free-text responses (3/26, 12%) to assess perceptions of changes to documentation. Some studies (3/26, 12%) conducted focus groups. In some studies (6/26, 23%), findings about documentation changes draw upon a broader dataset, while in others (20/26, 77%), they are supported only by individual quotes from practitioners. All of the studies with qualitative components (26/26, 100%) report changes in clinician-perceived note characteristics (such as length, comprehensibility, accuracy, objectivity, tone, and wording) or changes in content (candor, quality, and tacit knowledge) and functional aspects (notes as a working tool) due to ORA implementation. In contrast, in a few studies (6/26, 23%), some clinicians reported experiencing no changes in clinical documentation due to the introduction of ORA [38,49,54,55,57,61].

#### Linguistic Characteristics

##### Length

Similar to the objective changes, some clinicians reported a change in the length of their notes. In 2 studies, clinicians reported that their notes were lengthened by additional text [59] and clinically irrelevant information [42]. However, there were also reports suggesting the opposite: notes became shorter and more concise with the introduction of ORA, partly because hypothetical information was no longer included [68] (refer to the Loss of Tacit Knowledge section) and partly because of concerns about reputational risk [13] (refer to the Candor and also Influences on Documentation Practices section).

##### Comprehensibility

The comprehensibility of clinical notes appears to be related to their length. While a concise but accurate clinical note is easy for HCPs to understand and work with, patients often require longer explanations in clinical notes to fully understand their content. For example, Alpert et al [49] describe the challenge of composing notes that are both more comprehensible and less intimidating for patients (incorporating more paraphrases and explanations), while also limiting the number of words to ensure their usability in clinical practice. In many of the included studies, HCPs report being more aware and considerate in their writing after implementing ORA, with the goal of creating clinical notes that are more comprehensible and understandable [42,63,65,66]. To achieve this, clinicians reported writing in a clear, concise, and simple manner while avoiding ambiguous terminology [49,50,62,66-68]. Several qualitative studies reported that HCPs modified their use of medical terminology following the implementation of ORA, either by avoiding, reducing, or adapting it to make it more understandable to patients [20,42,49,62,63,68]. In addition, in 2 studies, HCPs reported a decrease in the use of abbreviations and Latin expressions [55,67]. In a recent study conducted by Keuper et al [41], 40.1% of clinicians reported adopting lay language after ORA implementation. Two studies reported a reduction in medical terminology, but HCPs expressed reluctance to completely eliminate it, recognizing its importance for communication with colleagues [66,68]. One study outlined that the described linguistic adjustments peaked shortly after the implementation of ORA but gradually decreased over time, resulting in clinicians reporting a return to their previous documentation practices [65]. Furthermore, 2 studies reported that some clinicians did not observe any effects of ORA on their writing style and continued to use medical terminology to ensure the integrity of clinical documentation [4,65].

##### Accuracy

Some qualitative studies (8/26, 31%) suggest that clinicians strive to document more accurately, factually, formally, precisely, and objectively when sharing clinical notes with patients [3,20,41,49,66-68], partly to mitigate potential misunderstandings with patients [20]. Other practitioners experience that the implementation of ORA leads to clinical documentation that is less accurate, less direct, and less objective, as the lack of correct medical terminology hinders the accurate communication of information to other providers while potentially introducing irrelevant clinical data [20,42,63]. Some studies (6/26, 23%) report that note accuracy after ORA implementation can be ensured by clinicians being more mindful in their documentation practices [3,20,41,49,66,68].

##### Mindful Tone and Wording

HCPs from 15 studies reported adjusting the tone and wording of clinical notes when patients were able to read them [3,4,14,17,39,42,49,51,52,54,55,57,58,63,66]. Many professionals reported being more careful and cautious in terms of tone and word choice when writing their clinical notes after the implementation of ORA [3,14,49,66]. They reported making efforts to write notes in a professional and respectful manner, acknowledging the patients’ identity and experience [58,66]. In addition, the implementation of ORA reportedly led to perceived changes in how sensitive clinical and social information was documented [42]: some clinicians reported refraining from using language that patients might perceive as critical, provocative, or offensive to avoid upsetting or angering them [39,42,51,54,55]. For instance, clinicians reported that they avoided using subjective terms such as “troubled,” “difficult,” “disruptive,” or “noncompliant” to describe patients’ conditions [4,42]. Potentially stigmatizing or hurtful descriptions (eg, obesity and mental health issues) were often reportedly excluded from the clinical notes or paraphrased [4,42,54,55]. However, Alpert et al [57] noted that even after the implementation of ORA, derogatory terms such as “fat” were still present in the clinical notes, causing distress to the patients. In 3 studies, practitioners emphasized that the adoption of patient-friendly, sensitive documentation (after the implementation of ORA) compromised the quality of the clinical notes, as described in more detail in the following section [4,17,52].

#### Note Content and Functionality

##### Candor

In many of the included qualitative studies, clinicians reported being less candid in their documentation or omitting information from the clinical notes. Percentages of clinicians who stated they were less candid in their documentation ranged from 15% to 52% [17,19,42,48,51,53-56,60,62,69]. Two studies showed that significantly more professionals expected to be less honest in their documentation due to ORA than they were when actually sharing their notes with patients [48,69].

Many clinicians reported becoming more selective in what they write [17,43,64], exercising greater caution in deciding what information to include [50,68], and reducing unnecessary detail in their clinical notes [14,39,60,66]. Numerous studies reported that clinicians tended to “safeguard” sensitive and potentially harmful information (eg, domestic violence) to protect patients from potential negative consequences. This may result in information being described more generally, censored, or hidden altogether from the clinical note [4,19,38,42-44,48,51,54-56,60-64,66-68]. In some cases, practitioners blocked patients’ access to their clinical notes [19,38] or used parallel documentation, referred to as “shadow records.” Shadow records refer to unofficial, private documentation maintained by clinicians in various formats, which is kept distinct from shared documentation [14,51]. Because of the reported changes in the level of candor, detail, and information included in the documentation, some clinicians criticized the decline in the quality and effectiveness of clinical notes [4,17,62,63].

Fewer clinicians reported that the ORA has led to more detailed and open documentation, typically to avoid patient complaints about missing information [3,65]. However, some studies suggest no change in content and candor: clinicians were already writing openly, honestly, and respectfully before ORA was implemented, perceiving that no further changes were required when records were shared with patients [3,4,58,63,68].

##### Loss of Tacit Knowledge

Some clinicians reported that they excluded certain information—often sensitive or not yet confirmed—from the clinical notes. Clinicians primarily avoided tentative differential diagnoses (especially in cases of suspected serious illness) [17,38,42,62,63,68]. However, they also reported withholding their own assessments and observations [17,54,62]. This included hypotheses, concerns, “gut feelings,” and speculative information that might be helpful to the next clinician treating the same patient [17,20,62,68]. In doing so, clinicians may be striving to avoid causing misunderstanding and confusion [17,20,63,68] or arousing fear and offense in patients [13,42,62], especially when there is no time or opportunity to thoroughly discuss the preliminary information with the patient [20,68].

##### Value as a Working and Therapy Tool

The changes described in the content and structure of clinical notes resulting from the use of ORA also affect how clinical notes may be used as work and therapy tools. Some clinicians argued that the clinical notes are no longer effective as a work tool and do not adequately serve their professional purpose when shared with patients. This is attributed to the omission of preliminary or sensitive information and a shift toward more descriptive but less rigorous documentation [17,62,63]. Some studies suggested that clinicians prefer to use clinical notes primarily in the traditional sense, either as a communication tool with colleagues and other providers [4,42,67,68] or as a work tool for themselves [4,38,62]. According to several clinicians, the changes in documentation caused by ORA hinder interdisciplinary communication within the team [42,62,64] as well as the personal use of notes (eg, as a personal reminder) [4,38,62], making the clinical note a less efficient and valuable working tool [39,42,69].

Other practitioners saw the adaptation of their writing as a new opportunity to expand the function of clinical notes as a therapeutic tool. By communicating directly with the patient through clinical notes, they could better engage patients by including self-care instructions or different types of reminders [42,63], highlighting the patient’s strength and progress, and reassuring the patient that their perspective is heard and understood [66]. The notes were used to emphasize important aspects of the consultation, clarify goals, and provide educational resources [66].

#### Influences on Documentation Practices

Two studies found that HCPs may feel uncertain or vulnerable when writing shared documentation [54,60]. Aiming to protect patients from adverse outcomes such as misunderstanding [20,49,63,66], anxiety, and confusion [17,42,63,64,68], they reported being more careful and guarded in their writing [17,49,51,63,67]. This was seemingly fueled by their desire to avoid being perceived as harsh, critical, or judgmental in their documentation [39,42,49]. In addition, clinicians were aware of the potential reputational risks, medicolegal concerns, and patient safety consequences associated with ORA, which accordingly influenced their documentation practices [13,20,60].

Finally, it should be noted that clinicians write with several target audiences in mind when ORA is available. HCPs were usually more considerate of the patient’s reception of the note, while still documenting appropriately for colleagues, health insurers, billing, and other stakeholders [3,4,17,42,44,49,51,52,56,57,59-62,65,66,68]. They must also consider the possibility of the so-called secret readers, such as relatives or caregivers, who may have access to the clinical notes without the clinician’s knowledge [3,42,52,60-62,64]. Therefore, the introduction of the ORA presents practitioners with the major challenge of writing a uniform note to serve multiple and sometimes conflicting needs.

## Discussion

### Principal Findings

This scoping review is the first to assess the potential for changes in documentation following patient ORA. Most of the included studies, which incorporated qualitative components, report changes in clinician-perceived note characteristics, such as length, comprehensibility, accuracy, objectivity, tone, and wording. In addition, these studies highlight changes in content, such as candor and quality as well as functional aspects, such as the role of notes as a working tool, following the implementation of ORA. Conversely, a minority of studies indicated that some clinicians reported no discernible changes in clinical documentation following the introduction of ORA [38,49,54,55,57,61]. Similar to the objective changes noted, some clinicians observed alterations in the length of their notes. In 2 studies, clinicians reported that their notes were longer [42,59]. However, contrasting subjective findings indicate the opposite effect [68]. Regarding the content of free-text notes, the objective evidence remains inconclusive. Generally, only minor to moderate alterations in the content of clinical notes are reported when shared with patients via the web [40,47]. Moreover, several studies using literacy metrics investigated changes in the readability and complexity of clinical notes. While Blok et al [45] and Kind et al [47] reported an increase in complexity and a decrease in readability, Rahimian et al [23] found a significant decrease in complexity and an increase in readability.

The mixed findings reported may reflect the diverse perspectives and experiences of the different individuals involved. Just as people communicate differently, they also approach documentation in unique ways, influenced by their background, experiences, and perceptions of transparency, privacy, and integrity. Various factors, such as personal attitudes, power dynamics, and professional habits, might further contribute to the variation in documentation practices. Given this diversity, the implementation of ORA will inevitably impact health care staff’s documentation practices differently. Some may view it as an opportunity to extend their caregiving beyond face-to-face interactions, using clinical notes as a therapeutic tool to better engage with patients [42,63], highlighting progress, clarifying goals, and offering reassurance through ORA [66]. Contrastingly, others grapple with ethical dilemmas regarding the inclusion or omission of certain information. Practitioners in 15 studies indicated that they modified the tone and language used in clinical notes when patients had access to them [3,4,14,17,39,42,49,51,52,54,55,57,58,63,66]. Following the introduction of ORA, numerous professionals adopted a more deliberate and cautious approach toward tone and language selection in clinical notes [3,14,49,66].

This variance in approaches underscores the need for flexibility and understanding within the health care setting. In essence, the following question arises: can a one-size-fits-all approach accommodate the diverse needs and perspectives present in health care? The answer remains uncertain, highlighting the complexities of standardizing practices across such a multifaceted sector. As the language, format, and content of clinical notes may evolve following ORA, assessing whether such changes yield benefits or pose risks is imperative.

O’Neill [70] contests the common belief that transparency and truth are inherently linked, asserting instead that they are fundamentally at odds. O’Neill [70] argues that transparency compels document writers to obscure genuine information or motives, crafting content deemed suitable for public consumption and thereby fostering deception. Worsening the argument by O’Neill [70], Nguyen [71] adds that the pressure to conform to public expectations through transparency may lead experts to compromise the integrity of their documentation, abandoning nuanced insights, tacit knowledge, and expert judgment. In several of the included qualitative studies, clinicians did express being less candid in their documentation or selectively omitting information from clinical notes due to patient access. Reported percentages of clinicians admitting to reduced candor in their documentation varied from 15% to 52% [17,19,42,48,51,53-56,60,62,69]. On the contrary, 2 studies found that more professionals expected to be less honest in their documentation due to ORA than those who actually were when sharing their notes [48,69]. Adding weight to the argument by Nguyen [71], practitioners experience that ORA implementation results in clinical documentation that is less accurate, less straightforward, and less objective; the absence of correct medical terminology hinders effective communication with other providers and possibly introduces irrelevant clinical data [20,42,63].

The increase in readability and note length following the implementation of ORA also suggests that HCPs may have started documenting in a manner they perceived to be more understandable or accessible to a broader audience, such as patients [23]. Numerous clinicians indicate increased selectiveness in their documentation [17,43,64], exercising caution in determining the information to incorporate [50,68], and trimming unnecessary details from their clinical notes [14,39,60,66]. In certain instances, practitioners went as far as to restrict patients’ access to their clinical notes [19,38] or resorted to parallel documentation, also known as a “shadow record” [14,51]. Restricting patients’ access to parts or all of their EHR may be justifiable in certain situations (such as suspected coercive access in the context of domestic abuse) and may therefore be acceptable clinical practice [72]. The reported changes in the level of candor, detail, and information in the documentation led some clinicians to critique a decline in the quality and effectiveness of clinical notes [4,17,62,63].

Fewer clinicians noted that ORA implementation resulted in more detailed and transparent documentation, often aiming to prevent patient complaints about missing information [3,65]. However, contrary to the theory of transparency and deception by O’Neill [70], certain studies suggest no alteration in content and candor due to ORA: clinicians had already been writing openly, honestly, and respectfully before the implementation of ORA; thus, no adjustments were deemed necessary when records were shared [3,4,58,63,68].

Numerous qualitative studies indicate that clinicians aim for greater accuracy, formality, precision, and objectivity when sharing clinical notes with patients [3,20,41,49,66-68]. In total, 6 studies indicate that clinicians can enhance note accuracy following ORA implementation by adopting more mindful documentation practices [3,20,41,49,66,68]. We can assert that ORA offers both advantages and disadvantages. They potentially enhance patient care while simultaneously posing risks regarding what information is omitted.

### Extended Care Through Documentation

Patient ORA satisfies the moral argument that the information belongs to the patient. It is even argued that when patients feel in more control of their care, they will take better care of themselves [73]. While it is acknowledged that all patients, including those with mental illness, have the right to access information about their health, this raises ethical questions about the implications of transparency in health care documentation. Here lies a definite complexity in the concept of care and conducting it ethically. Can ORA completely align with the medical “do no harm” principle? An often-overlooked risk linked to providing patients access to their medical records is the so-called secret readers—individuals other than the patients themselves who may access their medical records for both positive and negative reasons [3,42,52,59-62,64]. The possibility of secret readers may prompt physicians to self-censor in their documentation as a means of additional care and protection of patients considered vulnerable, such as children in families affected by domestic violence. Numerous studies have documented that clinicians frequently take measures to safeguard sensitive and potentially harmful information, such as instances of domestic violence, to shield patients from potential negative repercussions. Therefore, this often leads to describing information in more general terms or entirely concealing it from the clinical note [4,19,38,42-44,48,51,54-56,60-64,66-68]. Confidentiality in these fragile circumstances is paramount due to the potential escalation of abuse if a perpetrator discovers unwanted disclosure. ORA, therefore, heightens concerns about coercion and privacy breaches concerning issues of domestic violence and abuse. In established patient portals, such as the Swedish portal, safeguards have been implemented by excluding certain specifically tagged keywords, such as “risk for domestic violence,” from ORA [74].

### To Document or Not

When clinicians choose to exclude specific information, particularly sensitive or unconfirmed details such as provisional differential diagnoses [17,38,42,62,63,68] from shared clinical notes, it can be viewed as a form of practical care, aiming to prevent unnecessary worry or distress for the patient. Alongside these caregiving perspectives of omitting information due to ORA, a serious downside can arise when clinicians frequently refrain from including subjective assessments and observations [17,54,62]. This encompasses hypotheses, concerns, “gut feelings,” and speculative information that could help the next clinician treating the same patient [17,20,62,68]. In a health care context with high staff turnover, omitting information from a shared working document, although with the best intentions of care for the patient, could inevitably put the same patient at risk. Changes to documentation that are found to cause errors and lead to patient harm could also place clinicians at increased risk of malpractice [75]. By omitting this often tacit knowledge, clinicians aim to prevent misunderstanding and confusion [17,20,63,68] as well as avoid instilling fear and offense in patients [13,42,62], particularly when there is insufficient time or opportunity to thoroughly discuss preliminary information with the patient [20,68].

Research indicates that HCPs often experience feelings of insecurity and vulnerability when composing shared documentation [54,60], striving to shield patients from potential adverse outcomes, including misunderstanding [20,63,66], anxiety, and confusion [17,42,63,68]. Consequently, they tend to be more cautious and guarded in their writing [17,51,63,67]. This caution is further fueled by their desire to avoid being perceived as overly harsh, critical, or judgmental in their documentation [39,42].

### Changing Function of the EHR

The traditional function of the medical record has never been to inform, empower, or engage the patient; rather, its primary purpose has been to serve as a tool for HCPs to document clinical information. Several clinicians argue that the alterations in documentation induced by ORA hinder interdisciplinary communication within the team [42,62] and diminish the personal utility of notes (eg, as a personal reminder) [4,38,62], thereby reducing the effectiveness of clinical notes as a working tool. Certain clinicians contend that clinical notes are ineffective as a professional tool and fail to fulfill their intended purpose. This is attributed to the omission of preliminary or sensitive information and a transition toward more descriptive but less precise documentation [17,62,63]. Training on how to write clinical notes in the context of ORA could help HCPs navigate the changing role of the EHR. A recent survey of psychotherapy trainees found that 9 out of 10 believed that education on open notes should be part of the curriculum [76].

In an era of advancing digitization, ORA can be viewed as a burgeoning trend aimed at empowering patients through transparency, with medical documentation increasingly tailored for patients as one of the primary audiences. However, this shift may lead to deviations from the ethical principles that underpin patient care. It is important to recognize that not all aspects of health care can or should be shaped to be pleasing and empowering. Being a patient inherently involves vulnerability and a reliance on the expertise and trustworthiness of HCPs. Patients may feel objectified or reduced to a diagnostic label, seeking recognition of their individuality rather than being treated as a mere statistic or case study. However, in medical records, HCPs are not *describing* an individual; they are documenting a disease, which often causes confusion in a multipurpose document that is interpreted by various audiences. Research shows that clinicians opted not to use language that patients could interpret as critical, provocative, or offensive to prevent causing patients distress or frustration [39,42,51,54,55]. However, being a patient is ultimately intertwined with being vulnerable and distressed; this is essential and part of being in someone’s care, which ultimately leads to the patient being forced to trust and live through that vulnerability.

While ORA can facilitate patient engagement and transparency, it also introduces new considerations regarding data privacy, accuracy, and interpretation. Consequently, it is essential to recognize the limitations of digital documentation in fostering trust and prioritize cultivating trust through meaningful patient-HCP interactions. As seen in the included research, one reason clinicians omit preliminary findings in ORAs, in particular, is the lack of time to discuss them thoroughly with the patient [20,68].

### Limitations

The studies included in this scoping review have some notable limitations. The search for articles to be included in the review was conducted in the fall of 2023; therefore, the most recent evidence may not be included. Slightly less than half of the studies (19/42, 45%) were based on surveys, and it is unclear whether response bias may have affected the findings. In addition, studies reporting subjective changes in documentation were more prevalent than objective studies. Furthermore, some studies, particularly those using qualitative interviews, had relatively small sample sizes. Both these factors may affect the generalizability of the results. However, the inclusion of qualitative studies allows a more nuanced and comprehensive understanding of the results, and the studies with small sample sizes are counterbalanced by those with larger sample sizes.

In some studies, such as those conducted by Zellmer et al [56], clinicians were able to selectively choose which notes to share with patients and which to withhold. Assuming that particularly sensitive topics might have been avoided or not shared, this may influence the extent to which the documentation changed, as such topics especially require a patient-friendly adjustment of the clinical note.

Participating clinicians volunteered to participate in the studies and thus volunteered to share their clinical notes with patients. Therefore, it can be assumed that the clinicians had a positive attitude toward ORA or were at least interested in it (self-selection bias); critical voices may be underrepresented. However, as our results show a wide variation, it can be assumed that this is not the case.

### Conclusions

While there may be variations in the outcomes and attitudes among clinicians, it is evident that ORA does exert an influence on medical documentation practices. While it may not affect all health care staff uniformly, its effects are palpable for some, potentially influencing health care outcomes. Several included qualitative studies show that HCPs modify their use of medical terminology, either by avoiding, reducing, or adapting it to make it more understandable to patients [20,42,49,62,63,68]. This practical measure could indeed be seen as extended caregiving through documentation. In numerous of the included studies, professionals report being more aware and considerate in their writing after implementing ORA, with the goal of creating clinical notes that are more comprehensible and understandable [42,63,65,66]. Other patient benefits from ORA include clinicians’ attempts to write notes professionally and respectfully, acknowledging the patients’ identity and experience [58,66].

In contrast to the positive outcomes of documentation changes due to ORA, the presence of compensatory measures, such as parallel documentation [14,51] and restriction of patients’ access to their clinical notes [19,38], indicates a systemic issue, suggesting that current practices are not yet functioning optimally. Parallel documentation in this matter refers to shadow recording including unofficial, private documentation maintained by clinicians in various formats, which is kept distinct from shared documentation. Practical compensatory measures underscore deficiencies in the current system, wherein crucial information may be omitted or obscured. If an HCP, for example, withholds information out of fear or concerns regarding reputational risks [13], this might ultimately jeopardize the safety of their colleagues and delay proper patient care. The consequences of physicians omitting information that would benefit their colleagues in differing ways, even if only practiced by a minority, can have a cascading effect on patient care. Given that patient health care journeys are collaborative efforts involving multiple professionals, the impact of 1 note being influenced by ORA extends beyond individual patients and physicians. Such practices, whether they involve avoiding gut feelings or diverting critical information to less formal channels, jeopardize patient safety.

Finally, it should be noted that clinicians must write with multiple audiences in mind when implementing ORA. Clinicians were more considerate of the patient when writing the shared note, while still documenting appropriately for colleagues, health insurers, billing, and other stakeholders [3,4,17,42,44,49,51,52,56,57,59-62,65,66,68]. Therefore, the introduction of ORA presents practitioners with the major challenge of writing a uniform note. Many clinicians expressed a desire for system-level guidance regarding optimal documentation practices to mitigate potential negative outcomes for themselves or their patients. They emphasized the importance of training in recovery-oriented and strengths-focused treatment approaches, shifting from problem-focused thinking to a collaborative relationship with clients [60].

Using ORA effectively without omitting information does present a challenge. Future research must explore practical strategies regarding how ORA can be designed to navigate the complex surroundings of such a vital and multifaceted working document, ensuring ORA does not compromise the efficiency and security of care delivery, patients, or health care staff. To this end, we expect that future scoping reviews and empirical research will focus on the use of generative artificial intelligence in documentation practices [77,78]. There is already evidence that clinicians may be using these tools to assist with writing sensitive, understandable notes that patients will read [79]. It remains unclear whether such practices effectively meet the dual requirements of preserving documentation for clinicians while making the notes more understandable and empathetic for patients [80].

## Appendix

Multimedia Appendix 1 PRISMA-ScR (Preferred Reporting Items for Systematic Reviews and Meta-Analyses Extension for Scoping Reviews) checklist.

Multimedia Appendix 2 Search string.

Multimedia Appendix 3 Relevant data from included studies.

Multimedia Appendix 4 Mixed Methods Appraisal Tool grading.

## Abbreviations

EHR: electronic health record
HCP: health care professional
MMAT: Mixed Methods Appraisal Tool
ORA: online record access
PRISMA: Preferred Reporting Items for Systematic Reviews and Meta-Analyses
PRISMA-ScR: Preferred Reporting Items for Systematic Reviews and Meta-Analyses Extension for Scoping Reviews

## Data Availability

All data generated or analyzed during this study are included in this published article (and its supplementary information files). All studies analyzed during this study are publicly available and can be found via the following databases: APA PsycINFO, CINAHL, PubMed, and Web of Science Core Collection.
