# Supplementary material for: Impact of Patient Online Record Access on Documentation: Scoping Review
Source: J Med Internet Res. 2025 Feb 20;27:e64762. doi: 10.2196/64762 (PMC11888084; doi:10.2196/64762)
Supplement: Multimedia Appendix 2 [file jmir_v27i1e64762_app2.pdf]

Multimedia Appendix 2 Search String

CINAHL, 2023-07-31		
Search no	Search terms	Results
1	(MH "Patient Portals")	281
2	TI ("inpatient portal*" OR "open notes" OR opennotes OR PAEHR OR "patient portal*" OR "patient web portal*") OR AB ("inpatient portal*" OR "open notes" OR opennotes OR PAEHR OR "patient portal*" OR "patient web portal*")	1,193
3	#1 OR #2	1,308
4	(MH "Electronic Health Records")	28,806
5	TI ("clinic notes" OR "clinical notes" OR "progress notes" OR "doctors notes" OR EHR OR "health record*" OR "healthcare record*" OR "medical record*" OR "mental health notes" OR "patient record*" OR "psychiatric notes" OR "psychotherapy notes" OR "visit notes") OR AB ("clinic notes" OR "clinical notes" OR "progress notes" OR "doctors notes" OR EHR OR "health record*" OR "healthcare record*" OR "medical record*" OR "mental health notes" OR "patient record*" OR "psychiatric notes" OR "psychotherapy notes" OR "visit notes")	75,327
6	#4 OR #5	90,435
7	(MH "Patient Access to Records")	993
8	TI ("guardian access" OR "parental access" OR "parents access" OR "patient access*" OR "patients access*" OR "patient online access" OR "patients online access" OR "proxy access" OR "shared medical record*" OR "shared health record*") OR AB ("guardian access" OR "parental access" OR "parents access" OR "patient access*" OR "patients access*" OR "patient online access" OR "patients online access" OR "proxy access" OR "shared medical record*" OR "shared health record*")	4,198
9	#7 OR #8	5,056
10	#6 AND #9	926
11	#3 OR #10	2,032
12	(MH "Language+") OR (MH "Attitude+")	12,006
13	TI (accura* OR adopt* OR alter* OR ambigu* OR attitude* OR censor* OR change* OR changing OR characteristic* OR characters OR clarity OR completeness OR comprehend* OR comprehensib* OR comprehension* OR content* OR correctness OR dialog* OR difference* OR directness OR emotion* OR experience* OR express* OR implement* OR impression* OR inaccura* OR incomplete* OR incomprehen* OR incorrectness* OR intelligib* OR interpret* OR introduc* OR intuitive* OR	3,294,325

	language OR length OR linguistic* OR misconception* OR misinterpret* OR misread* OR misunderstand* OR modif* OR monolog* OR negative* OR pattern* OR perception* OR positive* OR postimplement* OR pronoun* OR readab* OR satisfact* OR simplicity OR style* OR terminolog* OR transparen* OR truthful* OR unambigu* OR understand* OR untruthful* OR veracity OR wordcount* OR words OR writing) OR AB (accura* OR adopt* OR alter* OR ambigu* OR attitude* OR censor* OR change* OR changing OR characteristic* OR characters OR clarity OR completeness OR comprehend* OR comprehensib* OR comprehension* OR content* OR correctness OR dialog* OR difference* OR directness OR emotion* OR experience* OR express* OR implement* OR impression* OR inaccura* OR incomplete* OR incomprehen* OR incorrectness* OR intelligib* OR interpret* OR introduc* OR intuitive* OR language OR length OR linguistic* OR misconception* OR misinterpret* OR misread* OR misunderstand* OR modif* OR monolog* OR negative* OR pattern* OR perception* OR positive* OR postimplement* OR pronoun* OR readab* OR satisfact* OR simplicity OR style* OR terminolog* OR transparen* OR truthful* OR unambigu* OR understand* OR untruthful* OR veracity OR wordcount* OR words OR writing)	
14	#12 OR #13	3,297,388
15	#11 AND #14	1,300
16	#15 *Narrow by Language: English*	1,295
17	#16 NOT ((ZT "commentary") OR (ZT "editorial") OR (ZT "newspaper") OR (ZT "opinion"))	1,261

PsycInfo, 2023-07-31		
Search no	Search terms	Results
1	TI ("inpatient portal*" OR "open notes" OR opennotes OR PAEHR OR "patient portal*" OR "patient web portal*") OR AB ("inpatient portal*" OR "open notes" OR opennotes OR PAEHR OR "patient portal*" OR "patient web portal*")	302
2	DE "Electronic Health Records"	2,095
3	TI ("clinic notes" OR "clinical notes" OR "progress notes" OR "doctors notes" OR EHR OR "health record*" OR "healthcare record*" OR "medical record*" OR "mental health notes" OR "patient record*" OR "psychiatric notes" OR "psychotherapy notes" OR "visit notes") OR AB ("clinic notes" OR "clinical notes" OR "progress notes" OR "doctors notes" OR EHR OR "health record*" OR "healthcare record*" OR "medical record*" OR "mental health notes" OR "patient record*" OR "psychiatric notes" OR "psychotherapy notes" OR "visit notes")	17,330
4	#2 OR #3	17,784
5	TI ("guardian access" OR "parental access" OR "parents access" OR "patient access*" OR "patients access*" OR "patient online access" OR "patients online access" OR "proxy access" OR "shared medical record*" OR "shared health record*") OR AB ("guardian access" OR "parental access" OR "parents access" OR "patient access*" OR "patients access*" OR "patient online access" OR "patients online access" OR "proxy access" OR "shared medical record*" OR "shared health record*")	1,127
6	#4 AND #5	132
7	#1 OR #6	399
8	DE "Adolescent Attitudes" OR DE "Adult Attitudes" OR DE "Attitudes" OR DE "Child Attitudes" OR DE "Client Attitudes" OR DE "Comprehension" OR DE "Counselor Attitudes" OR DE "Language" OR DE "Linguistics" OR DE "Parental Attitudes" OR DE "Phrases" OR DE "Pronouns" OR DE "Psychologist Attitudes" OR DE "Readability" OR DE "Sentences" OR DE "Terminology" OR DE "Vocabulary" OR DE "Written Language"	229,724
9	TI (accura* OR adopt* OR alter* OR ambigu* OR attitude* OR censor* OR change* OR changing OR characteristic* OR characters OR clarity OR completeness OR comprehend* OR comprehensib* OR comprehension* OR content* OR correctness OR dialog* OR difference* OR directness OR emotion* OR experience* OR express* OR implement* OR impression* OR inaccura* OR incomplete* OR incomprehen* OR incorrectness* OR intelligib* OR interpret* OR introduc* OR intuitive* OR language OR length OR linguistic* OR misconception* OR misinterpret* OR misread* OR misunderstand* OR modif* OR	3,998,661

	monolog* OR negative* OR pattern* OR perception* OR positive* OR postimplement* OR pronoun* OR readab* OR satisfact* OR simplicity OR style* OR terminolog* OR transparen* OR truthful* OR unambigu* OR understand* OR untruthful* OR veracity OR wordcount* OR words OR writing) OR AB (accura* OR adopt* OR alter* OR ambigu* OR attitude* OR censor* OR change* OR changing OR characteristic* OR characters OR clarity OR completeness OR comprehend* OR comprehensib* OR comprehension* OR content* OR correctness OR dialog* OR difference* OR directness OR emotion* OR experience* OR express* OR implement* OR impression* OR inaccura* OR incomplete* OR incomprehen* OR incorrectness* OR intelligib* OR interpret* OR introduc* OR intuitive* OR language OR length OR linguistic* OR misconception* OR misinterpret* OR misread* OR misunderstand* OR modif* OR monolog* OR negative* OR pattern* OR perception* OR positive* OR postimplement* OR pronoun* OR readab* OR satisfact* OR simplicity OR style* OR terminolog* OR transparen* OR truthful* OR unambigu* OR understand* OR untruthful* OR veracity OR wordcount* OR words OR writing)	
10	#8 OR #9	4,017,854
11	#7 AND #10	366
12	#11 AND *Language: English*	363
13	#12 NOT ((ZZ "column/opinion") OR (ZZ "comment/reply") OR (ZZ "editorial"))	351

PubMed, 2023-07-31		
Search no	Search terms	Results
1	"Patient Portals"[MeSH]	734
2	"inpatient portal*"[Title/Abstract] OR "open notes"[Title/Abstract] OR opennotes[Title/Abstract] OR PAEHR[Title/Abstract] OR "patient portal*"[Title/Abstract] OR "patient web portal*"[Title/Abstract]	1,905
3	#1 OR #2	2,050
4	"Electronic Health Records"[MeSH]	27,841
5	"clinic notes"[Title/Abstract] OR "clinical notes"[Title/Abstract] OR "progress notes"[Title/Abstract] OR "doctors notes"[Title/Abstract] OR EHR[Title/Abstract] OR "health record*"[Title/Abstract] OR "healthcare record*"[Title/Abstract] OR "medical record*"[Title/Abstract] OR "mental health notes"[Title/Abstract] OR "patient record*"[Title/Abstract] OR "psychiatric notes"[Title/Abstract] OR "psychotherapy notes"[Title/Abstract] OR "visit notes"[Title/Abstract]	195,624
6	#4 OR #5	204,431
7	"Patient Access to Records"[Mesh]	1,160
8	"guardian access"[Title/Abstract] OR "parental access"[Title/Abstract] OR "parents access"[Title/Abstract] OR "patient access*"[Title/Abstract] OR "patients access*"[Title/Abstract] OR "patient online access"[Title/Abstract] OR "patients online access"[Title/Abstract] OR "proxy access"[Title/Abstract] OR "shared medical record*"[Title/Abstract] OR "shared health record*"[Title/Abstract]	6,144
9	#7 OR #8	7,151
10	#6 AND #9	1,217
11	#3 OR #10	2,979
12	"Language"[Mesh] OR "Attitude"[Mesh] OR "Comprehension"[Mesh]	874,916
13	accura*[Title/Abstract] OR adopt*[Title/Abstract] OR alter*[Title/Abstract] OR ambigu*[Title/Abstract] OR attitude*[Title/Abstract] OR censor*[Title/Abstract] OR change*[Title/Abstract] OR changing[Title/Abstract] OR characteristic*[Title/Abstract] OR characters[Title/Abstract] OR clarity[Title/Abstract] OR completeness[Title/Abstract] OR comprehend*[Title/Abstract] OR comprehensib*[Title/Abstract] OR comprehension*[Title/Abstract] OR content*[Title/Abstract] OR correctness[Title/Abstract] OR dialog*[Title/Abstract] OR difference*[Title/Abstract] OR directness[Title/Abstract] OR emotion*[Title/Abstract] OR experience*[Title/Abstract] OR express*[Title/Abstract] OR implement*[Title/Abstract] OR	17,557,195

	impression*[Title/Abstract] OR inaccura*[Title/Abstract] OR incomplete*[Title/Abstract] OR incomprehen*[Title/Abstract] OR incorrectness*[Title/Abstract] OR intelligib*[Title/Abstract] OR interpret*[Title/Abstract] OR introduc*[Title/Abstract] OR intuitive*[Title/Abstract] OR language[Title/Abstract] OR length[Title/Abstract] OR linguistic*[Title/Abstract] OR misconception*[Title/Abstract] OR misinterpret*[Title/Abstract] OR misread*[Title/Abstract] OR misunderstand*[Title/Abstract] OR modif*[Title/Abstract] OR monolog*[Title/Abstract] OR negative*[Title/Abstract] OR pattern*[Title/Abstract] OR perception*[Title/Abstract] OR positive*[Title/Abstract] OR postimplement*[Title/Abstract] OR pronoun*[Title/Abstract] OR readab*[Title/Abstract] OR satisfact*[Title/Abstract] OR simplicity[Title/Abstract] OR style*[Title/Abstract] OR terminolog*[Title/Abstract] OR transparen*[Title/Abstract] OR truthful*[Title/Abstract] OR unambigu*[Title/Abstract] OR understand*[Title/Abstract] OR untruthful*[Title/Abstract] OR veracity[Title/Abstract] OR wordcount*[Title/Abstract] OR words[Title/Abstract] OR writing[Title/Abstract]	
14	#12 OR #13	17,841,601
15	#11 AND #14	2,443
16	#15 AND "english"[Language]	2,405
17	#16 NOT ("address"[Publication Type] OR "comment"[Publication Type] OR "editorial"[Publication Type] OR "news"[Publication Type])	2,364

UPPSALA UNIVERSITET

Uppsala University Library

Web of Science Core Collection, 2023-07-31		
Search no	Search terms	Results
1	TS=("inpatient portal*" OR "open notes" OR opennotes OR PAEHR OR "patient portal*" OR "patient web portal*")	2,080
2	TS=("clinic notes" OR "clinical notes" OR "progress notes" OR "doctors notes" OR EHR OR "health record*" OR "healthcare record*" OR "medical record*" OR "mental health notes" OR "patient record*" OR "psychiatric notes" OR "psychotherapy notes" OR "visit notes")	187,981
3	TS=("guardian access" OR "parental access" OR "parents access" OR "patient access*" OR "patients access*" OR "patient online access" OR "patients online access" OR "proxy access" OR "shared medical record*" OR "shared health record*")	6,768
4	#2 AND #3	844
5	#1 OR #4	2,693
6	TS=(accura* OR adopt* OR alter* OR ambigu* OR attitude* OR censor* OR change* OR changing OR characteristic* OR characters OR clarity OR completeness OR comprehend* OR comprehensib* OR comprehension* OR content* OR correctness OR dialog* OR difference* OR directness OR emotion* OR experience* OR express* OR implement* OR impression* OR inaccura* OR incomplete* OR incomprehen* OR incorrectness* OR intelligib* OR interpret* OR introduc* OR intuitive* OR language OR length OR linguistic* OR misconception* OR misinterpret* OR misread* OR misunderstand* OR modif* OR monolog* OR negative* OR pattern* OR perception* OR positive* OR postimplement* OR pronoun* OR readab* OR satisfact* OR simplicity OR style* OR terminolog* OR transparen* OR truthful* OR unambigu* OR understand* OR untruthful* OR veracity OR wordcount* OR words OR writing)	34,391,479
7	#5 AND #6	2,133
8	#7 AND (LA==("ENGLISH"))	2,102
9	#8 NOT (DT==("EDITORIAL MATERIAL"))	2,060
