# Supplementary material for: Impact of Patient Online Record Access on Documentation: Scoping Review
Source: J Med Internet Res. 2025 Feb 20;27:e64762. doi: 10.2196/64762 (PMC11888084; doi:10.2196/64762)
Supplement: Multimedia Appendix 4 [file jmir_v27i1e64762_app4.pdf]

Multimedia Appendix 4. MMAT ratings for each study. (1…yes; 0…no; ?...can’t tell)

Studies		Criteria from the Mixed Methods Appraisal Tool																								
**Authors**	**Ref.-ID**	1.1	1.2	1.3	1.4	1.5	2.1	2.2	2.3	2.4	2.5	3.1	3.2	3.3	3.4	3.5	4.1	4.2	4.3	4.4	4.5	5.1	5.2	5.3	5.4	5.5
Ålander et al., 2015	1																1	1	1	1	1					
Alpert et al., 2016	2	1	1	1	1	1																				
Alpert et al., 2019a	3																					1	1	1	1	1
Alpert et al., 2019b	4																					1	1	1	1	1
Blease et al., 2023	5	1	1	1	1	1																				
Blok et al., 2021	6											1	1	1	1	1										
Cajander et al., 2018	7	1	1	1	1	1																				
Chimowitz et al., 2020	8	1	1	1	1	1																				
Choi et al., 2022	9																					1	1	1	1	1
Crucefix et al., 2021	10	1	1	1	1	1																				
Davidge et al., 2023	11	1	1	1	1	1																				
Delbanco et al., 2012	12																1	1	1	1	1					
Denneson et al., 2017	13	1	1	1	1	1																				
DesRoches et al., 2020	14																1	1	1	1	1					
Dobscha et al., 2016	15																1	1	1	1	1					
Drinkwater et al., 2017	16	1	1	1	1	1																				
Erlingsdóttir et al., 2019	17	1	1	1	1	1																				
Grünloh et al., 2016	18	1	1	1	1	1																				
Holmgren et al., 2022	19											1	1	1	1	1										
Jain et al., 2017	20																1	1	1	1	1					
Johansen et al., 2019	21																					1	1	1	1	1
Keuper et al., 2023	22																1	1	1	0	1					
Kind et al., 2011	23											1	1	1	1	1										
King et al., 2017	24																					1	1	1	1	1
Leonard et al., 2023	25																1	1	1	0	1					
Moll et al., 2020a	26																1	0	1	1	1					
Moll et al., 2020b	27	1	?	1	0	1																				
Muli et al., 2022	28	1	1	1	1	1																				
Oster et al., 2015	29											1	1	1	1	1										
Petersson et al., 2018	30																					1	1	1	0	1
Pisciotta et al., 2019	31	1	1	1	1	1																				
Rahimian et al., 2019	32																1	1	1	1	1					
Rahimian et al., 2021	33																1	1	1	1	1					
Ralston et al., 2021	34											1	1	0	1	1										
Richards et al., 2021	35																					1	1	1	1	1

Smaradottir, 2018	36	1	1	1	0	1																				
Turner et al., 2023	37	1	1	1	1	1																				
Turvey et al., 2022	38																					1	1	1	0	1
Walker et al., 2011	39											1	1	1	0	1										
Wass et al., 2018	40																					1	1	1	1	1
Zanaboni et al., 2022	41	1	1	1	1	1																				
Zellmer et al., 2021	42																					1	1	1	0	0

List of references

1. Ålander T, Scandurra I. Experiences of Healthcare Professionals to the Introduction in Sweden of a Public eHealth Service: Patients' Online Access to their Electronic Health Records. Stud Health Technol Inform books.google.com; 2015;216:153–157. PMID:26262029

2. Alpert JM, Krist AH, Aycock RA, Kreps GL. Applying Multiple Methods to Comprehensively Evaluate a Patient Portal's Effectiveness to Convey Information to Patients. J Med Internet Res 2016 May 17;18(5):e112. PMID:27188953

3. Alpert JM, Morris BB, Thomson MD, Matin K, Geyer CE, Brown RF. OpenNotes in oncology: oncologists' perceptions and a baseline of the content and style of their clinician notes. Transl Behav Med academic.oup.com; 2019 Mar 1;9(2):347–356. PMID:29596633

4. Alpert JM, Morris BB, Thomson MD, Matin K, Sabo RT, Brown RF. Patient access to clinical notes in oncology: A mixed method analysis of oncologists' attitudes and linguistic characteristics towards notes. Patient Educ Couns 2019 Oct 1;102(10):1917–1924.

5. Blease C, Torous J, Dong Z, Davidge G, DesRoches C, Kharko A, Turner A, Jones R, Hägglund M, McMillan B. Patient Online Record Access in English Primary Care: Qualitative Survey Study of General Practitioners' Views. J Med Internet Res 2023 Feb 22;25:e43496. PMID:36811939

6. Blok AC, Amante DJ, Hogan TP, Sadasivam RS, Shimada SL, Woods S, Nazi KM, Houston TK. Impact of Patient Access to Online VA Notes on Healthcare Utilization and Clinician Documentation: a Retrospective Cohort Study. J Gen Intern Med 2021 Mar;36(3):592–599. PMID:33443693

7. Cajander Å, Moll J, Englund S, Hansman A. Medical Records Online for Patients and Effects on the Work Environment of Nurses. Stud Health Technol Inform books.google.com; 2018;247:271–275. PMID:29677965

8. Chimowitz H, O'Neill S, Leveille S, Welch K, Walker J. Sharing Psychotherapy Notes with Patients: Therapists' Attitudes and Experiences. Soc Work academic.oup.com; 2020 Apr 1;65(2):159–168. PMID:32236447

9. Choi HH, Kotsenas AL, Chen JV, Bronsky C, Roth CJ, Kohli MD. Multi-institutional Experience with Patient Image Access Through Electronic Health Record Patient Portals. J Digit Imaging 2022 Apr;35(2):320–326. PMID:35022926

10. Crucefix AL, Fleming APL, Lebus CS, Slowther A-M, Fritz Z. Sharing a written medical summary with patients on the post-admission ward round: A qualitative study of

clinician and patient experience. J Eval Clin Pract Wiley; 2021 Dec;27(6):1235–1242. PMID:33960593

11. Davidge G, Brown L, Lyons M, Blease C, French D, van Staa T, McMillan B. Primary care staff's views and experience of patients' online access to their electronic health record: a qualitative exploration. Br J Gen Pract 2023 Jun;73(731):e418–e426. PMID:37068967

12. Delbanco T, Walker J, Bell SK, Darer JD, Elmore JG, Farag N, Feldman HJ, Mejilla R, Ngo L, Ralston JD, Ross SE, Trivedi N, Vodicka E, Leveille SG. Inviting patients to read their doctors' notes: a quasi-experimental study and a look ahead. Ann Intern Med acpjournals.org; 2012 Oct 2;157(7):461–470. PMID:23027317

13. Denneson LM, Cromer R, Williams HB, Pisciotta M, Dobscha SK. A Qualitative Analysis of How Online Access to Mental Health Notes Is Changing Clinician Perceptions of Power and the Therapeutic Relationship. J Med Internet Res jmir.org; 2017 Jun 14;19(6):e208. PMID:28615152

14. DesRoches CM, Leveille S, Bell SK, Dong ZJ, Elmore JG, Fernandez L, Harcourt K, Fitzgerald P, Payne TH, Stametz R, Delbanco T, Walker J. The Views and Experiences of Clinicians Sharing Medical Record Notes With Patients. JAMA Netw Open jamanetwork.com; 2020 Mar 2;3(3):e201753. PMID:32219406

15. Dobscha SK, Denneson LM, Jacobson LE, Williams HB, Cromer R, Woods S. VA mental health clinician experiences and attitudes toward OpenNotes. Gen Hosp Psychiatry 2016 Jan;38:89–93. PMID:26380876

16. Drinkwater J, Stanley N, Szilassy E, Larkins C, Hester M, Feder G. Juggling confidentiality and safety: a qualitative study of how general practice clinicians document domestic violence in families with children. Br J Gen Pract 2017 Jun;67(659):e437–e444. PMID:28137783

17. Erlingsdóttir G, Petersson L, Jonnergård K. A Theoretical Twist on the Transparency of Open Notes: Qualitative Analysis of Health Care Professionals' Free-Text Answers. J Med Internet Res jmir.org; 2019 Sep 25;21(9):e14347. PMID:31573905

18. Grünloh C, Cajander Å, Myreteg G. "The record is our work tool!"—Physicians' framing of a patient portal in Sweden. J Med Internet Res JMIR Publications Inc.; 2016 Jun 27;18(6):e167.

19. Holmgren AJ, Apathy NC. Assessing the impact of patient access to clinical notes on clinician EHR documentation. J Am Med Inform Assoc 2022 Sep 12;29(10):1733–1736. PMID:35831954

20. Jain SK, Rahimian M, Joyce RM, Zerillo JA, Warner JL. Using network graphs to visualize changing documentation styles in an oncology practice before and after opennotes implementation. 2017 IEEE Workshop on Visual Analytics in Healthcare (VAHC) IEEE; 2017. p. 62–68.

21. Johansen MA, Kummervold PE, Sørensen T, Zanaboni P. Health Professionals' Experience with Patients Accessing Their Electronic Health Records: Results from an Online Survey. Stud Health Technol Inform ebooks.iospress.nl; 2019 Aug 21;264:504–508. PMID:31437974

22. Keuper J, Batenburg R, van Tuyl L, Verheij R. General Practices' Experiences With Patients' Web-Based Access to Medical Records: Survey Study. J Med Internet Res 2023 Apr 7;25:e41832. PMID:37027195

23. Kind EA, Fowles JB, Craft CE, Kind AC, Richter SA. No change in physician dictation patterns when visit notes are made available online for patients. Mayo Clin Proc

2011 May;86(5):397–405. PMID:21531883

24. King G, Maxwell J, Karmali A, Hagens S, Pinto M, Williams L, Adamson K. Connecting Families to Their Health Record and Care Team: The Use, Utility, and Impact of a Client/Family Health Portal at a Children's Rehabilitation Hospital. J Med Internet Res 2017 Apr 6;19(4):e97. PMID:28385680

25. Leonard SM, Zackula R, Wilcher J. Attitudes and Experiences of Clinicians After Mandated Implementation of Open Notes by the 21st Century Cures Act: Survey Study. J Med Internet Res 2023 Feb 28;25:e42021. PMID:36853747

26. Moll J, Cajander Å. Oncology health-care professionals' perceived effects of patient accessible electronic health records 6 years after launch: A survey study at a major university hospital in Sweden. Health Informatics J journals.sagepub.com; 2020 Jun;26(2):1392–1403. PMID:31621459

27. Moll J, Cajander Å. On Patient Accessible Electronic Health Records and the Experienced Effect on the Work Environment of Nurses. Stud Health Technol Inform 2020 Jun 16;270:1021–1025. PMID:32570536

28. Muli I, Scandurra I, Cajander Å, Hägglund M. Healthcare Professionals' Experiences of the Work Environment After Patients' Access to Their Electronic Health Records - A Qualitative Study in Primary Care. Stud Health Technol Inform 2022 May 25;294:530–534. PMID:35612136

29. Oster NV, Jackson SL, Dhanireddy S, Mejilla R, Ralston JD, Leveille S, Delbanco T, Walker JD, Bell SK, Elmore JG. Patient Access to Online Visit Notes: Perceptions of Doctors and Patients at an Urban HIV/AIDS Clinic. J Int Assoc Provid AIDS Care 2015 Jul-Aug;14(4):306–312. PMID:24729072

30. Petersson L, Erlingsdóttir G. Open Notes in Swedish Psychiatric Care (Part 2): Survey Among Psychiatric Care Professionals. JMIR Mental Health 2018;5(2).

31. Pisciotta M, Denneson LM, Williams HB, Woods S, Tuepker A, Dobscha SK. Providing mental health care in the context of online mental health notes: advice from patients and mental health clinicians. J Ment Health 2019 Feb;28(1):64–70. PMID:30468100

32. Rahimian M, Warner JL, Jain SK, Davis RB, Zerillo JA, Joyce RM. Significant and Distinctive n-Grams in Oncology Notes: A Text-Mining Method to Analyze the Effect of OpenNotes on Clinical Documentation. JCO Clin Cancer Inform ascopubs.org; 2019 Jun;3:1–9. PMID:31184919

33. Rahimian M, Warner JL, Salmi L, Rosenbloom ST, Davis RB, Joyce RM. Open notes sounds great, but will a provider's documentation change? An exploratory study of the effect of open notes on oncology documentation. JAMIA Open academic.oup.com; 2021 Jul;4(3):ooab051. PMID:34661067

34. Ralston JD, Yu O, Penfold RB, Gundersen G, Ramaprasan A, Schartz EM. Changes in Clinician Attitudes Toward Sharing Visit Notes: Surveys Pre-and Post-Implementation. J Gen Intern Med Springer; 2021 Nov;36(11):3330–3336. PMID:33886028

35. Richards S, Carlson K, Matthias T, Birge J. Perception versus reality: Does provider documentation behavior change when clinic notes are shared electronically with patients? Int J Med Inform 2021 Jan;145:104304. PMID:33129123

36. Smaradottir BF. Patient Accessible Electronic Health Records: Impacts on Nursing Documentation Practices at a University Hospital. Stud Health Technol Inform 2018;250:14–18. PMID:29857356

37. Turner A, Morris R, McDonagh L, Hamilton F, Blake S, Farr M, Stevenson F, Banks J, Atherton H, Rakhra D, Lasseter G, Feder G, Ziebland S, Hyde E, Powell J, Horwood J. Unintended consequences of patient online access to health records: a qualitative study in UK primary care. Br J Gen Pract bjgp.org; 2023 Jan;73(726):e67–e74. PMID:36316163

38. Turvey CL, Fuhrmeister LA, Klein DM, Moeckli J, Howren MB, Chasco EE. Patient and Provider Experience of Electronic Patient Portals and Secure Messaging in Mental Health Treatment. Telemed J E Health 2022 Feb;28(2):189–198. PMID:33887164

39. Walker J, Leveille SG, Ngo L, Vodicka E, Darer JD, Dhanireddy S, Elmore JG, Feldman HJ, Lichtenfeld MJ, Oster N, Ralston JD, Ross SE, Delbanco T. Inviting patients to read their doctors' notes: patients and doctors look ahead: patient and physician surveys. Ann Intern Med acpjournals.org; 2011 Dec 20;155(12):811–819. PMID:22184688

40. Wass S, Vimarlund V. Same, same but different: Perceptions of patients' online access to electronic health records among healthcare professionals. Health Informatics J journals.sagepub.com; 2019 Dec;25(4):1538–1548. PMID:29874962

41. Zanaboni P, Kristiansen E, Lintvedt O, Wynn R, Johansen MA, Sørensen T, Fagerlund AJ. Impact on patient-provider relationship and documentation practices when mental health patients access their electronic health records online: a qualitative study among health professionals in an outpatient setting. BMC Psychiatry 2022 Jul 28;22(1):508. PMID:35902841

42. Zellmer BM, Nacht CL, Coller RJ, Hoonakker PLT, Smith CA, Sklansky DJ, Dean SM, Smith W, Sprackling CM, Ehlenfeldt BD, Kelly MM. BedsideNotes: Sharing Physicians' Notes With Parents During Hospitalization. Hosp Pediatr publications.aap.org; 2021 May;11(5):503–508. PMID:33795371
